# Clinical findings and risk factors to oral squamous cell carcinoma in young patients: A 12-year retrospective analysis

**DOI:** 10.4317/medoral.20770

**Published:** 2016-01-31

**Authors:** Hellen-Bandeira-de-Pontes Santos, Thayana-Karla-Guerra dos Santos, Alexandre-Rolim Paz, Yuri-Wanderley Cavalcanti, Cassiano-Francisco-Weege Nonaka, Gustavo-Pina Godoy, Pollianna-Muniz Alves

**Affiliations:** 1 MsC in Dentistry, Post-graduate Program of Dentistry, State University of Paraíba, Campina Grande, Paraíba, Brazil; 2 MsC in Dentistry, Post-graduate Program of Dentistry, Federal University of Paraíba, João Pessoa, Paraíba, Brazil; 3 Professor, Department of Pathology, Federal University of Paraíba, João Pessoa, Paraíba, Brazil; 4 Post-Doctoral Fellow at School of Dentistry, State University of Paraíba, Campina Grande, Paraíba, Brazil; 5 Professor, Post-graduate Program of Dentistry, State University of Paraíba, Campina Grande, Paraíba, Brazil

## Abstract

**Background:**

In recent years have been observed an increased incidence of OSCC in young individuals. Based on this, the aim this study was to describe the clinical characteristics of all cases of OSCC in younger patients, diagnosed in two oncology referral hospitals, at the northeast region of Brazil within a 12-year period.

**Material and Methods:**

Data regarding general characteristics of patients (age, gender and tobacco and/or alcohol habits) and information about the lesions (tumor location, size, regional lymph node metastasis, distant metastasis and clinical stage) were submitted to descriptive and inferential analysis. Statistical analysis included Chi-square and Fisher’s exact tests (*P*<0.05).

**Results:**

Out of 2311 registered cases of OSCC, 76 (3.3%) corresponded to OSCC in patients under 45 years old. Most of them were male (n=62, 81.6%) and tobacco and/or alcohol users (n=40, 52.8%). The most frequent site was the tongue (n=31, 40.8%), with predominance of cases classified at advanced clinical stage (III and IV, n = 46, 60.5%). The advanced stage of OSCC (III and IV) was statistically associated with male gender (*P*=0.035), lower education level (*P*=0.007), intraoral site(*P*<0.001), presence of pain symptomatology (*P*=0.006), and consumption of tobacco and/or alcohol (*P*=0.001).

**Conclusions:**

The profile of OSCC in young patients resembles to the commonly characteristics reported for overall population. The late diagnosis in young patients usually results in poor prognosis, associated with gender, harmful habits and tumor location. Although prevalence is low, stimulus to prevention and to early diagnosis should be addressed to young individuals exposed to risk factors.

**Key words:**Squamous cell carcinoma, head and neck neoplasms, risk factors, young patients, prognosis.

## Introduction

Oral Squamous Cell Carcinoma (OSCC) is the most frequent malignant neoplasm of oral cavity corresponding to 80 to 90% of all malignancies (1). OSCC mainly affects men within their sixth and seventh decades of life. An increasing incidence of OSCC among individuals younger than 45 years old has been observed in recent decades, representing approximately 4% to 13% of all cases of OSCC (2-8). The tongue is the anatomical site more frequently affected and it is usually associated with alcohol and tobacco use (2,3). Additionally, intensive exposure to sunlight is the main etiological factor for the squamous cell carcinoma at the lower lip (4).

Since young individuals are exposed for a short period of time to significant risk factors (i.e.: extended exposition to sunlight and consumption of tobacco and/or alcohol); studies have suggested that the etiology of OSCC differs between younger and elderly patients (7,9-12). In addition, some studies have suggested that younger patients with OSCC are likely nonsmokers and nondrinkers (10,11,13). Therefore, the literature has pointed out that other factors might be associated with OSCC in young population, such as genetic predisposition, immunological and nutritional alterations and infection by HPV (7,13,14). However, this relationship is still not well established.

In terms of biological behavior and clinical prognosis, evidences suggest that OSCC in younger patients have increased aggressiveness compared to those affecting elderly patients (4,5,10,15,16). However, the definition of prognosis is still hard to determine and improved prognostic characteristics would be clinically useful to determine the biological aggressiveness of OSCC, for both younger and elder patients.

Regional lymph node metastasis, tumor location and TNM classification of malignant tumors (TNM) has been cited as prognostic indicators (17). However, there are scarce studies in the literature regarding the clinical prognostic factors of OSCC in young patients worldwide. Based on that, our study aimed to examine all cases of OSCC in young patients, diagnosed in two oncology hospitals within a 12-year period.

## Material and Methods

- Study design 

A 12-year retrospective and retrolective analysis was conducted with the clinical records of all individuals diagnosed with OSCC, within two oncology referral hospitals, at the northeast region of Brazil. The ethical committee in research of the State University of Paraiba has approved the present investigation under protocol number 163.442. To access the records of all OSCC patients, the principals of the hospitals provided an informed consent form authorizing the use of data for research purposes.

The study population consisted of all cases of OSCC diagnosed in patients under 45 years old, at the oncology referral hospitals. The patients’ age at the first OSCC diagnostic determined their allocation within the study population. Recurrent OSCC cases under management and those undergoing radiotherapy or chemotherapy were excluded from the study.

- Variables and data collection methods

General and medical records of individuals included gender, age, race, level of education and tobacco and/or alcohol habits. Additional information regarding the characteristics of the lesion was also considered, including: tumor location, pain symptomatology, treatment, size of the tumor, presence of regional lymph node metastasis, distant metastasis and clinical stage. The evaluation regarding the clinical stage of lesions was in accordance with the Union for International Cancer Control (UICC) classification of malignant tumors described in 2002. This classification can be used in a way to determine the prognostic of lesions and survival rate of patients (7,18).

- Statistical analysis

Data was analyzed descriptively by the calculation of proportions. Statistical analysis included associations between the characteristics of individuals (gender, education level, pain symptomatology and tobacco and/or alcohol habits) and lesions (size, tumor location, regional lymph node metastasis, distant metastasis and clinical stage). Statistical associations were performed using Chi-square and Fisher’s exact tests, at 5% significance level (*P*<0.05).

## Results

Out of 2311 registered cases of OSCC, 76 (3.3%) corresponded to patients under 45 years old. The proportion of OSCC in younger patients, per year, was also determined using the total number of cases within 12 years, as shown in  Table 1. Prevalence varied between 2.63% and 17.11% and bigger proportion was found in 2010, showing that prevalence was not regular along the years.

**Table 1:**
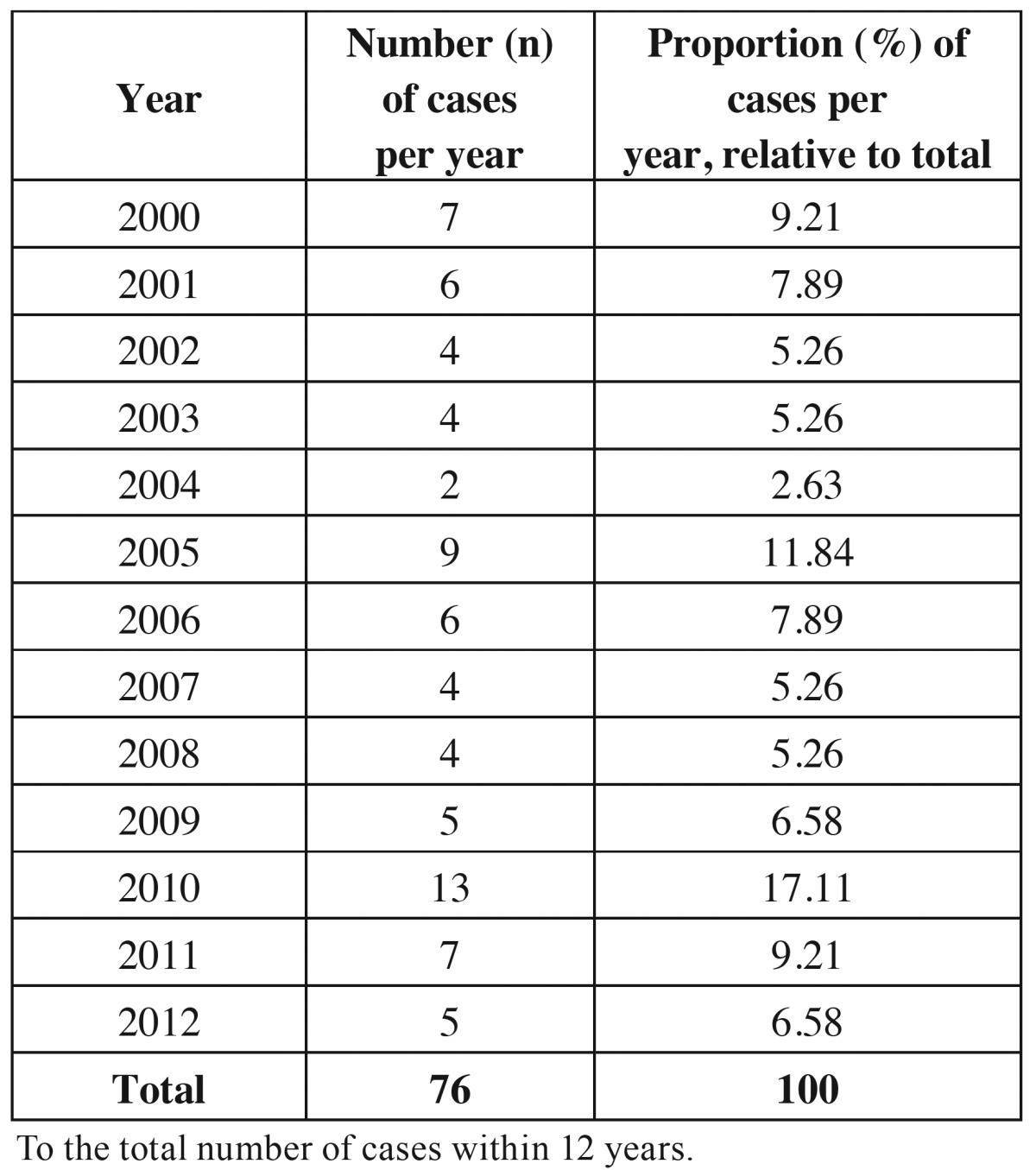
Prevalence of OSCC cases in young patients per year and proportion relative to the total number of cases within 12 years.

Within the young population studied, age varied between 22 and 45 years with median of 38.38 ± 6.47 years old. OSCC affected predominantly male (n = 62, 81.6%), in a ratio of 4.42:1 between men and women. Most of patients declared themselves as “non-white” (n =40, 52.7%). The level of education among the studied individuals was considered low (n= 43, 56.6%).

Simultaneous use of alcohol and tobacco was the most frequent habit reported (n= 28, 36.8%). With regards to primary tumor location, OSCC affected predominantly tongue (n = 31, 40.8%) and lower lip (n = 24, 31.6%) ( Table 2). The later corresponded to the only group of tumors located extra-orally. The pain symptomatology was present in 69.7% (n=53) of patients. With regards to the treatment performed, surgery was frequently associated with radiotherapy and chemotherapy (n=36, 47.4%) ( Table 2).

**Table 2:**
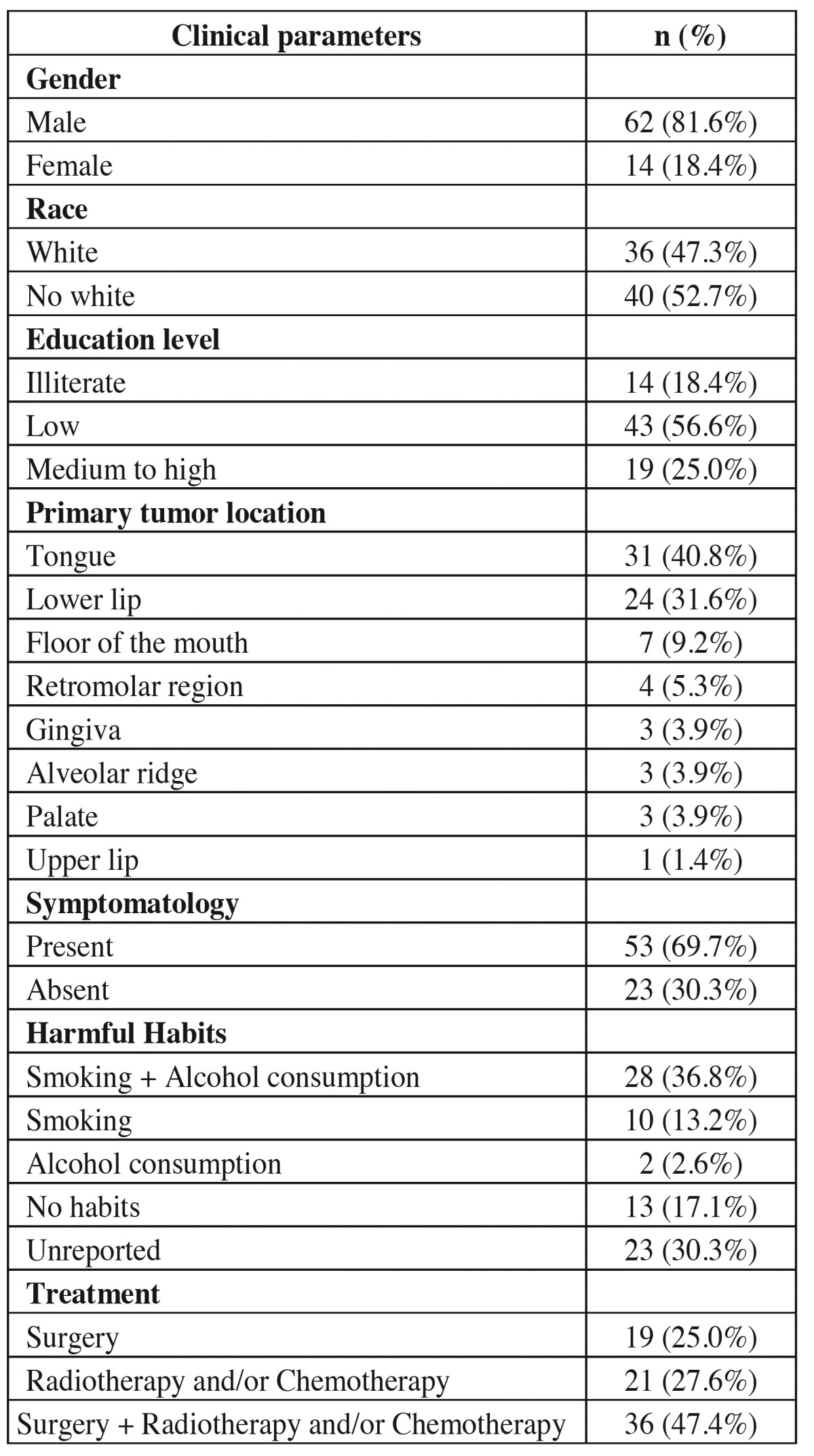
Absolute and relative distributions of OSCC cases in young patients, according to clinical parameters.

Based on TNM classification, tumor size “T1” (n = 27, 35.5%) and absence of regional lymph node metastasis “N0” (n = 40, 52.6%) were more frequently reported. Distant metastasis was absent within the population studied (n = 76, 100%) ( Table 3).

**Table 3:**
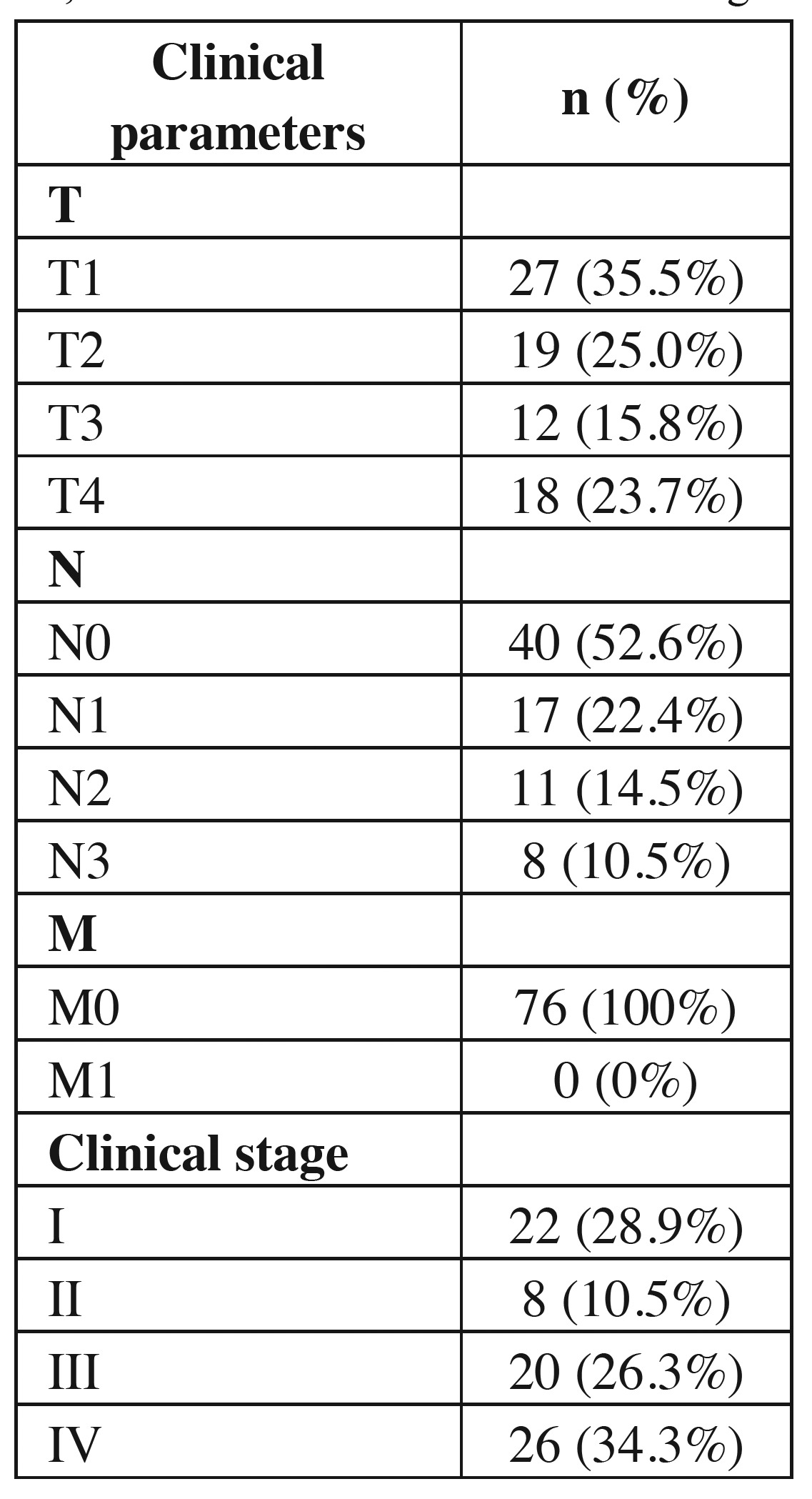
Absolute and relative distributions of OSCC cases in young patients, according to tumor size, nodal metastasis, distant metastasis and clinical stage.

Lesions at advanced clinical stage (III and IV, n = 46, 60.5%) were recurrently found at intraoral sites (n=38, 82.6%), frequently affecting male individuals (n = 41, 89.1%), smokers and drinkers (n= 31, 77.5%), illiterate (n=11, 78.6%) and patients reporting pain symptomatology (n=35, 66.0%). Therefore, clinical stage was statistically associated with gender (*P*=0.035), education level (*P*=0.007) and tumor location (*P*<0.001). Similarly, advanced clinical stages were associated with the presence of tobacco and alcohol habits (*P*=0.001) and pain symptomatology (*P*=0.006) ( Table 4).

**Table 4:**
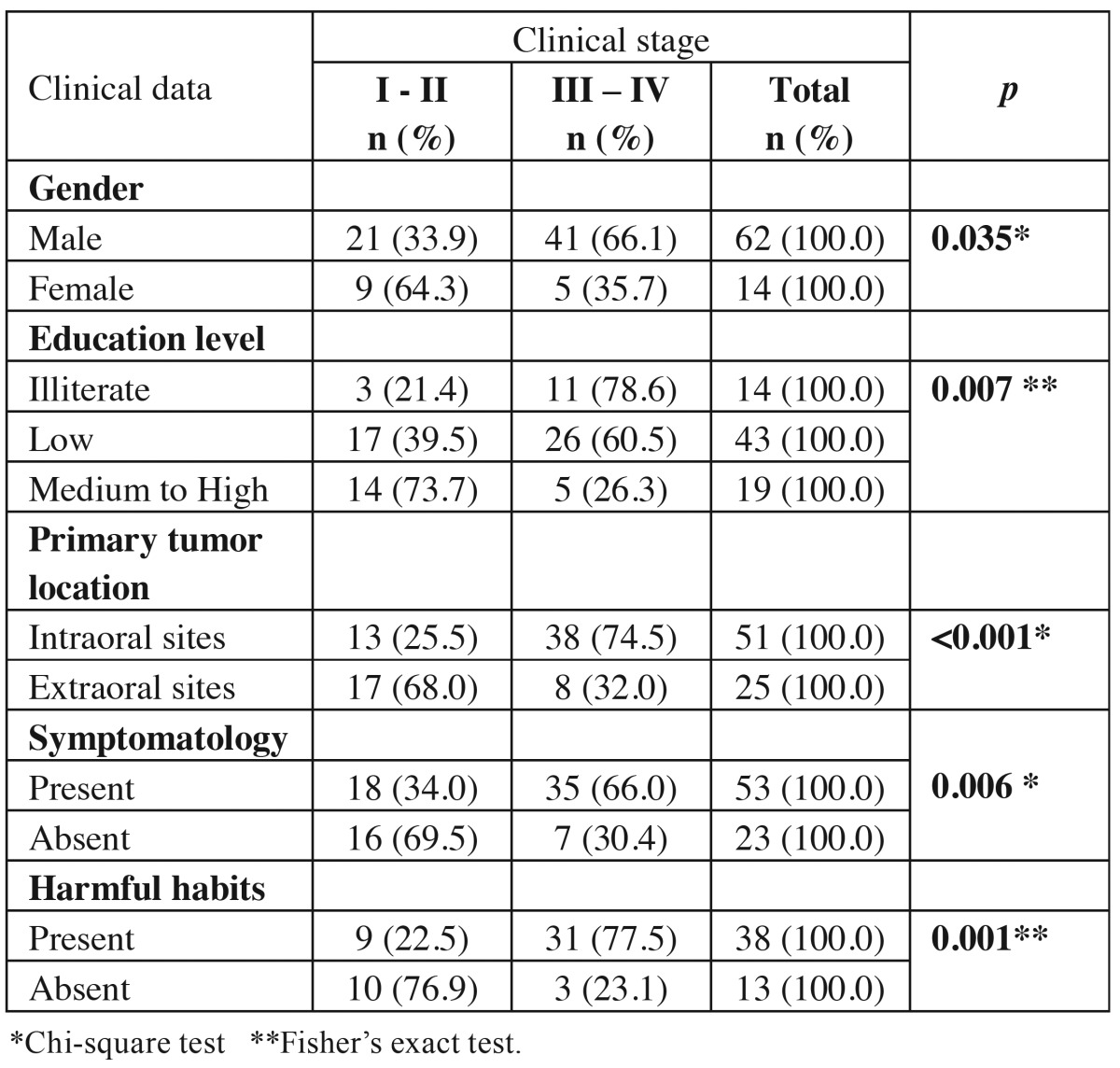
Absolute and relative distributions of OSCC cases in young patients, regarding the association between clinical data and clinical stage.

## Discussion

Although OSCC is recognized as malignant neoplasm affecting mainly elderly patients, the incidence of OSCC in individuals under 45 years old has increased substantially within the past few years (4-7,10,12,16). Studies have suggested that OSCC develops a more aggressive behavior, with poor prognosis, among young patients (5,19,20). In this census study, the analysis of clinical records from two oncology referral hospitals in the northeast region Brazil has pointed out important risk factors implicated with the etiology and pathogenicity of OSCC among young individuals, resulting in a consistent epidemiological profile for this age group.

Out of the 2311 cases of OSCC, 3.3% were diagnosed in young patients, as similarly shown by the literature (6,12). However, greater prevalence of OSCC among young individuals was informed by other studies, with prevalence ranging from 12% to 14% (4,8,10). In the present study, the prevalence of OSCC varied between 2.63% and 17.11%, not showing any pattern of manifestation along the years. Regional, socioeconomic and cultural characteristics of different populations may explain the discrepancy found in the reported prevalence of OSCC by various studies with diverse methodological design.

The population included in the present study has cultural habit to consume alcohol and tobacco since first years of adulthood. This aspect might be associated with increased prevalence of OSCC in tongue (40.8%). Additionally, the population of present study is exposed to high incidence of sunlight radiation, due to the proximity of the region to the equatorial line. This fact may be associated high prevalence of OSCC at the lower lip (31.6%). Tumors affecting the tongue, the floor of the mouth and the lower lip have been included as the most frequent location on OSCC independently of age group (1,4,11,20). Furthermore, individuals with diagnostic of OSCC in the present study were predominantly men with low educational level. As demonstrated by the statistical analysis performed, those characteristics were significantly associated with advanced clinical stage of OSCC.

With regards to gender, higher prevalence of OSCC has been frequently reported among male individuals, independently of the age group (8,10,11,21). Our study corroborates the literature, since 81.6% (n=62) of OSCC occurred in men (4,6,8,10,11,22,23). In contrast, other studies have found higher prevalence of OSCC in young female patients (12,24). Different population characteristics such as genetic predisposition, altered immune and hormonal modulations and infections by HPV may explain the differences within the prevalence between genders (5,10).

The etiology of OSCC is considered complex due to its multifactorial characteristic (4,8,10,11,15,16,20-27). Tobacco and alcohol consumption have been firmly stated as important risk factors for OSCC among elderly people. However, the ability of those habits develop OSCC in youth population is questionable (4,10,15,28).

In the present study, tobacco and/or alcohol consumption was frequently presented (75.4%), which reflects a cultural and social habit frequently present during early adulthood in developing countries such as Brazil (4). The current report confirmed not only the association between OSCC and tobacco and/or alcohol consumption, but also suggested the relation with advanced clinical stage. Additional data relative to tobacco and/or alcohol habits, as age of starting and quantity of consumption per day was not available, therefore we declare the lack of this relevant information as a limitation of our study. Of course, others risk factors might be associated with occurrence of OSCC among young patients (e.g. hereditary predisposition, syndromes, bad nutrition, immune-suppression and HPV infection) (4,9-11,14,20,25-29). However, those aspects were not object of the present investigation.

The prevalence of OSCC at advanced stages (III and IV) was high (60.6%) and corroborated other investigations (4,5). Besides that, advanced stages (III and IV) of OSCC in young patients were statistically associated with male gender, lower education level, intraoral tumors, pain symptomatology and consumption of tobacco and/or alcohol. Analogous relationship has been reported to OSCC among elderly people (2,3). Therefore, based in our results, we suggested that these conditions must be considered as relevant risk factors to the development of OSCC in young patients. Poor prognosis and lower survival rates are expected among patients under those risk factors.

In contrast with the worst prognosis of intraoral lesions, extra-oral tumors (detected predominantly at lower lip) were diagnosed at initial stages. Besides the lower aggressiveness (30), those lesions are anatomically located at easily detectable sites, which allows earlier diagnoses. Therefore, due to early diagnostic, a better prognosis and extended survival rate would be expected for those patients. Studies regarding the etiology and pathogenicity of OSCC at lower lip of young patients are scarce within the literature.

Early diagnostic of carcinogenic lesions has been reported among young (27) and female populations (29). Possibly, those individuals have greater ability to detect alterations within their anatomical structures, being also more concerned about their own healthcare. The present investigation has shown that young women had lower prevalence of OSCC and also detected those alterations within early stages of development (*P*=0.035). In general, the prevalence of OSCC among young patients was low in comparison to literature; however, preventive measures should be implemented and stimulus to early diagnosis must be enforced considering the risk factors pointed out by the present study.

Within the limitations of the present study, the following conclusions can be drawn. The clinical and epidemiological profile of young patients affected by OSCC is similar to that reported to elderly population. Higher prevalence and poor prognosis of OSCC among young patients are associated with male gender, lower educational level, and pain symptomatology. Furthermore, consumption of alcohol and tobacco, as well as tumors located intraorally are significantly associated with high prevalence and poor prognosis of OSCC among young individuals. Further investigations should consider other multi-centric approaches to elucidate the etiology and biological behavior of OSCC in young patients.
